# Please be Honest and Provide Evidence: Deterrents of Deception in an Online Insurance Fraud Context

**DOI:** 10.1002/acp.3252

**Published:** 2016-07-19

**Authors:** Sharon Leal, Aldert Vrij, Galit Nahari, Samantha Mann

**Affiliations:** ^1^ Department of Psychology University of Portsmouth Portsmouth UK; ^2^ Department of Criminology Bar‐Ilan University Ramat Gan Israel

## Abstract

The present experiment examined whether people could be deterred from lying in an online insurance claim setting. A total of 96 participants were asked to submit a theft insurance claim. Reflecting real life, submitting a claim that went beyond the actual costs of the stolen items was associated with advantages and disadvantages. Two deterrence factors were introduced: asking claimants to provide evidence that they actually owned the stolen items (Evidence Instruction, often used by insurers) and asking participants to read out before starting to submit the claim that they will be truthful (Honesty Statement, not often used by insurers). We also examined at what stage of the interview claimants embedded their lies in their otherwise truthful stories. The honesty statement but not the evidence instruction made claimants more honest, and participants lied more as the interview progressed. © 2016 The Authors Applied Cognitive Psychology Published by John Wiley & Sons Ltd.

For many decades, forensic deception research primarily focused on police–suspect interviews (Vrij & Granhag, 2012, 2014). Recently, it has been argued that other domains neglected in deception research are equally important, such as lying in intelligence interviews (Brandon, 2011; Loftus, 2011). Another important but neglected area is lie detection in financial settings. For example, the Association of British Insurers (ABI, 2009) reported that 20% of general insurance holders in the UK said that they would consider making an exaggerated or completely made up insurance claim in the future. The ABI further estimated that undetected insurance claims fraud totals £1.3bn a year in the UK in 2013, adding £50 to the annual costs of individual policyholders (BBC, 2014, http://www.bbc.co.uk/news/business-27608316).

Lie detection is difficult. A meta‐analysis of lie detection research including 206 documents and 24 483 observers revealed that observers classify correctly, on average, 54% of truth tellers and liars (Bond & DePaulo, 2006). This is a low percentage given that 50% accuracy can be expected just by flipping a coin. Other meta‐analyses have shown that individual differences in the ability to detect deceit are minute and that the poor performance occurs across various groups of observers (Aamodt & Custer, 2006; Bond & DePaulo, 2008).

If lie detection is so difficult, can we then perhaps deter people from lying? The research in this area is scarce, but recently, Van 't Veer, Stel, and van Beest (2014) found that people can indeed be deterred from lying when ‘cognitive load’ is imposed on them, that is, when the task of lying is made more difficult. In their study, participants rolled a die and reported the outcomes while under high cognitive load (memorising a string of eight letters) or low cognitive load (two letters to memorise). The higher the score the participants reported, the more money they could earn. The reported outcomes under low cognitive load were significantly higher than those under high cognitive load, suggesting that the participants under low cognitive load lied more than the participants under high cognitive load.

In the present experiment, we examined whether we could deter people from lying when completing an insurance claim regarding the theft of items. We know from life experience that insurance companies ask claimants to provide evidence that they actually owned the items they claimed for, ideally by showing the receipt or alternatively by providing a photo depicting the stolen item in their home. This policy makes sense as it gives insurers insight into the costs and ownership of the claimed items. Despite understanding the logic behind this policy, we have doubt that this evidence factor is effective in deterring people from lying. The instruction does little more than asking the claimant something they are already inclined to do. Claimants (honest and fraudulent alike) will understand that an insurer is more likely to pay if they provide evidence. Honest claimants are therefore likely to hand over the evidence if they have it. Liars typically tell a mixture of truths and lies (Leins, Fisher, & Ross, 2013; Nahari, Vrij, & Fisher, 2014). In an insurance context, this would mean exaggerating, in part, true claim rather than fabricating the entire claim. They then too are likely to submit evidence for at least some items they will claim for.

Perhaps another approach will work: To invite the claimant prior to starting to complete the form to declare that she or he will complete the form truthfully. The theoretical rationale for this ‘honesty’ factor is that it will focus the claimant on him or herself while reading out this declaration, which will stimulate self‐awareness. In turn, self‐awareness initiates an automatic comparison of the self against ethical and moral standards (Duval & Wicklund, 1972). Because people want to perceive themselves as moral (Aquino & Reed, 2002), self‐awareness makes someone behave accordingly to their moral standards and ethical goals (Baumeister & Heatherton, 1996), and avoiding being dishonest could be the result.

Research has shown the efficacy of the honesty factor in deterring people from lying. In two studies (a field study and an experiment), participants signed a written honesty statement prior to completing a form ‘I promise that the information I am providing is true’ or did so at the end of the form or not at all (Shu, Mazar, Gino, Ariely, & Bazerman, 2012). In the ‘honesty statement prior to the form condition’, participants in the field study admitted to have driven more miles in their car in the previous year (an acknowledgement that results in a higher premium), and participants in the laboratory experiment claimed less income and less travel expenses than participants in the other conditions (see also Ariely [2012] for a discussion of the findings).

Providing such an honesty statement in advance also raised the saliency to moral standards. Participants were presented with a list of word fragments and asked to fill in the blanks to make complete words by using the first word that came to mind (Shu et al., 2012). Three of these fragments (_ _ R A L, _ I _ _ _ E, and E _ _ _ C _ _) could potentially be completed by words related to ethics (moral, virtue and ethical). Those in the honesty statement condition came up with more words indicating moral standards (*M* = 1.40) than a control group (*M* = 0.87). Although insurers and tax authorities typically ask claimants to sign an honesty statement, they ask them to do this *after* the claimant completed the form rather than before (Shu et al., 2012). They do this for legal reasons as the statement and signature explicitly links the claimant with the form she or he just completed. The honesty declaration, however, only yielded an effect if it is made before completing the claim (Shu et al., 2012).

In the present experiment, participants took part in an online, verbal, automated insurance claim setting similar to an automated, verbal, phone call system frequently used by the financial industry (e.g. banks and insurance companies). In the experiment, we introduced the evidence instruction factor that insurers typically use (‘provide evidence’) and the honesty statement factor that they typically do not use (asking participants to declare that they will be honest before starting to complete the form). We predicted that the honesty statement would deter people from lying more than the providing evidence instruction (Hypothesis 1).

Apart from the deterrence of deceit, we also examined the moment when people lie. When being fraudulent while completing an insurance claim, people could lie during the entire claim or could tell a mixture of truths and lies, for example, by reporting some items that were really stolen but then to add to the list some made up items to boost their claim. Also, in other situations (e.g. job interviews and intelligence interviews), people could tell a mixture of truths and lies. A relevant question hereby is when people are most likely to lie when they report a mixture of truths and lies. This is relevant, because if an investigator knows when someone is most likely (or least likely) to lie, it could help him or her in detecting these lies. To our knowledge, the issue of when people lie when they tell a mixture of truths and lies has never been examined.

We thought it to be likely that most people will not start an interview with a lie. Most people probably first would like to familiarise themselves with the situation to see how the investigator responds or what the lie detection tool (if used) looks like. Another reason to postpone lying is to gain trust from the investigator. Indeed, once people are trustworthy, they will put less effort in lie detection and, consequently, liars have more chance to get away with their lies (Levine & McCornack, 1992). We therefore predicted that most participants would not lie when claiming the first item (Hypothesis 2). We also expected a linear trend with an increasing amount of lies told as the interview progresses (Hypothesis 3).

To sum up, the current study differs from Shu et al. (2012) in two ways. First, we examined the relative efficacy of two deterrence factors in one experiment (evidence and honesty), whereas Shu et al. only examined honesty. Second, we examined at which stage of the claim people are more likely to lie.

## Method

### Participants

A total of 96 participants (40 men and 56 women) took part. Participants ages ranged from 18 to 67 years with an average age of *M* = 29.23 years (*SD* = 12.22, 95% CI [3.16, 4.32]). No significant difference in the distribution of gender, *X* ^2^(3, 96) = 2.10, *p* = .56, Φ = 0.15, or in age, *F*(3, 764) = 1.75, *p* = 1.63, *η*_p_^2^ = 5.24, occurred across the four experimental conditions.

### Procedure

Participants were recruited via an advert on the university intranet and advertisement leaflets. The advert explained that the experiment would require participants to make a ‘convincing insurance claim’. On arrival at the Psychology department, participants were informed that they would read a short vignette about a mock burglary and that their task would be to make a convincing online insurance claim on behalf of two students (Rosie and Jack) who ostensibly had eight items stolen from their apartment. (Because participants did not experience the burglary themselves, we chose for the option to let them claim on behalf of someone else.) Once participants had read the burglary vignette, they received instructions outlining the advantages of telling the truth or lying. With telling the truth, that is, claiming back the actual value of the items stolen, they would earn £5; with increasing the actual claim value with £80, they would earn £10; and with increasing the actual claim value with £120, they would earn £15. In addition, they could earn more money if they made the claim convincingly. The most convincing truth teller could win an extra £50, and the two highest claims presented convincingly could win an extra £100 and £75. Therefore, a truth teller could potentially earn (with winnings) £55; a convincing liar could potentially earn £115 (with winnings), but an unconvincing liar could only earn a maximum of £15. This reflects real life in which insurance fraud could lead to gains. In reality, all participants were paid £15 for their time (even when they did not increase the actual claim), all truth tellers were entered into the £50 draw and all liars were entered into the £75 and £100 draw.

To ensure that participants fully understood the instructions, the experimenter emphasised to participants that it would be totally up to them to decide to lie or tell the truth and that, as in real life, there were potential benefits associated with their decision to lie. Participants were then given a printed list of the eight items with the actual cost of the item next to it (Laptop £370, Kindle £110, X‐box £350, iPod £200, DVD player £75, iPad £450, MacBook £900 and iPhone £600.) Additionally, participants were given varying degrees of evidence that Rosie and Jack owned the items, broken down as follows: (i) receipt and a photograph for the Kindle and Laptop, labelled ‘Receipt + photo’ items below; (ii) receipt only for the X‐box and iPod, labelled ‘receipt’ items below; (iii) photograph only for the iPad and DVD player, labelled ‘photo’ items below; and (iv) no evidence at all for the iPhone and MacBook, labelled ‘no evidence’ items below. Participants were informed that the varying evidence also reflected real life, in that claimants do not always have receipts and photographs for every item stolen.

All participants were told that they would take part in an online, verbal, automated insurance claim setting similar to an automated, verbal, phone call system used by the financial industry, but that instead of being verbally guided by a voice on the phone, they would be guided via the computer programme. Participants were required to respond to questions verbally, and they were informed that their verbal responses would be recorded and that these would later be judged for plausibility/likelihood of truthfulness by two independent raters.

Participants were then given time alone in a room with the list of items, the actual costs of the items and the available evidence for the items. They were provided with paper and informed that they could use their phones to search on the Internet if they wanted to. They were also told that apart from claiming for the eight items, which were actually stolen, they could fabricate claims for additional items, including cash. They were told to exit the room and tell the experimenter when they felt that they were ready to go into the cubicle and complete the online claim. All participants were informed that because they were not being tested on memory, they would be able to take the list of items, actual costs of the items, evidence and any notes they had made into the cubicle with them. Participants spent approximately 20 minutes preparing before exiting the room.

They then went into a cubicle where the computer screen was set up to look like an insurance company home claims page. It was explained to participants that the programme would ask about non‐cash items and cash money stolen and that non‐cash items referred to anything stolen that was not cash money. The basic programme was devised to verbally ask participants to claim for each item and record all the verbal responses given. Participants were guided through the programme with prompts such as ‘claim for first non‐cash item’ and then ‘claim another non‐cash item’ or ‘finish non‐cash item claim’. This allowed participants not only to claim for the eight items as required but also to add as many items as they wanted to. Once they went on to the next item, they could not go back to a previous item to change their claim. After they had clicked ‘finish non‐cash item claim’, they were asked if any cash was stolen in the burglary and if so, how much money, before being required to ‘submit final claim’. This basic programme was adjusted according to the two experimental factors ‘evidence instruction’ (absent versus present) and ‘honesty statement’ (absent versus present).

### Evidence instruction manipulation

In the evidence instruction absent condition, before participants started entering their claims, the interviewer's voice said: ‘Shortly, I will ask you to individually describe each non‐cash item you are claiming for. Please include the cost and condition of each item in your description’. Once the participant clicked start in this condition, a voice said ‘Please give a full description and cost of the item you are claiming for’. When the participant clicked ‘finish non‐cash items’, the voice said ‘Please tell us if any cash was stolen in the burglary and if so how much was taken’.

In the evidence instruction present condition, before participants started entering their claims, the interviewer's voice said in addition to what was said in the evidence instruction absent condition: ‘Additionally it is important that you provide evidence that the claimant actually owned the item, if you cannot do this then please explain in as much detail as possible why no evidence of ownership exists’. Once the participant clicked ‘start’ in this condition, the interviewer's voice then said ‘Please give a full description and cost of the item you are claiming for, including evidence that the claimant owned the item’, and this was repeated for every non‐cash item they claimed for. When the participant clicked ‘finish non‐cash items’, the interviewer's voice said ‘Please tell us if any cash was stolen in the burglary and if so how much was taken and why the money was there? Again, please include evidence that the claimant would have had that amount of cash in the apartment’.

### Honesty statement manipulation

In the honesty statement present condition, before starting entering their claims, participants were told that it is vital to us (the insurance company) that they would provide the information to the best of their knowledge and as truthful as it can be. They were asked to read out the following statement that appeared on the screen: ‘Hello my name is (please read out your name) and I state that the information I will give regarding this claim will be totally truthful to the best of my knowledge’ followed by the interviewer's voice saying ‘Thank you so much for your honesty, please click to claim for your first item’. In the honesty statement absent condition, no information was given.

After completing the online claim, participants filled out a post‐claim questionnaire. The questionnaire asked participants through an open‐ended question to indicate why they presented the items in the order they did. Each participant was then de‐briefed as to the purpose of the experiment and given £15 for taking part.

### Coding of online verbal responses

For each participant, all verbal responses were transcribed. From these transcripts, we calculated the order in which the items were claimed for, the amount of money claimed for each item and also the amount of cash claimed. In the Results section, we present the following sets of variables:

1. Amount of cash claimed, which was divided into seven variables: (i) ‘Receipt + Photo’; (ii) ‘Receipt’; (iii) ‘Photo’; (iv) ‘No Evidence’; (v) ‘new items added’; (vi) ‘cash’; and (vii) the total of i to vi. For variables i to iv, we recorded the amount of cash someone over‐claimed or under‐claimed. Thus, since the actual costs of the Macbook was £900, a claim of £950 would result in a score of £50, and a claim of £850 would result in a score of −£50. (Although we gave under‐claiming the costs a negative £ score, we did not code this as a lie, because this is not a type of lie an insurance company is worried about [as it works in their advantage]). For ‘new items added’, we recorded the total amount of money (in £) claimed for all the added items combined and for ‘cash’, we recorded the total amount of cash (in £) claimed.
2. When the first lie was told: For each participant, we recorded when the first lie was told. If the first item claimed was a lie (either a newly added item or an exaggeration of the costs of one of the eight stolen items), a score of ‘1’ was given; if the first lie was told in the second item claimed, a score of ‘2’ was given; if the first lie was told in the eight item claimed, a score of ‘8’ was given; and so forth. A missing value was entered for the participants who did not lie (*n* = 8). This variable could therefore reach from ‘1’ to ‘8’.
3. How many lies told in each of the first eight items claimed: For the first eight items claimed, we calculated the percentage of participants who lied during that item.

### Coding of open‐ended post‐questionnaire questions

The open‐ended responses from the post‐claim questionnaire were transcribed, and the experimenter devised categories to explain the strategies provided by participants. Each of the three established categories was put into a copy of Table 4 and given to an independent rater to code responses from all 96 participants. To ascertain inter‐rater reliability, a second independent coder rated 20 of the statements. Inter‐rater reliability between the two coders was ICC = 1.00 for the random strategy, ICC = 1.00 for the gain trust strategy and ICC = 0.89 for the third category.

### Ethics statement

Ethical approval for the study was gained through the University ethics committee in line with the British Psychological Society guidelines. Participants provided their written informed consent to participate in the study.

## Results

### Total number of lies told

The total number of lies told was *M* = 3.29, *SD* = 2.76. An ANOVA utilising a 2 (evidence instruction: absent versus present) × 2 (honesty statement: absent versus present) between‐subjects design and the number of lies as dependent variable revealed a main effect for evidence instruction, *F*(1, 92) = 10.989, *p* < .001, *d* = 0.72. Those who did not receive an evidence instruction told more lies (*M* = 4.19, *SD* = 3.28, 95% CI [3.44, 4.94]) than those who did receive this instruction (*M* = 2.40, *SD* = 1.72, 95% CI [1.67, 3.17]). The honesty statement main effect, *F*(1, 92) = .335, *p* = .564, *d* = 0.12, and the evidence instruction × honesty statement interaction effect, *F*(1, 92) = 2.068, *p* = .154, *η*_p_^2^ = 0.022, were not significant.

Eight participants did not lie. They were equally distributed over the evidence instruction absent and present conditions (*n* = 4 in each condition); they were also equally distributed in the honesty statement present (*n* = 5) and absent (*n* = 3) conditions, *X* ^2^(1, 96) = .459, *p* = .498, Φ = 0.069.

### The amount of money (£) claimed

First, we analysed how much extra money (on top of the actual costs of the eight stolen items) was claimed for each of the six types of items presented in Table 1. An ANOVA with Type of Item as within‐subjects factor revealed a significant effect, *F*(5, 91) = 6.12, *p* < .001, *η*_p_^2^ = 0.25. Simple effect tests revealed that the lowest amounts of extra money were claimed for the stolen items with evidence, followed by the stolen items without evidence. Most extra money was made by adding items to the list of stolen items and by claiming that cash money was stolen.

**Table 1:** Amount of extra money (on top of the actual costs of the items) in £ claimed

	*M*	*SD*	CI
Photo + receipt	16.44^a^	80.80	0.07, 32.81
Photo	22.09^a^	72.38	7.43, 36.76
Receipt	16.54^a^	63.84	3.60, 29.47
No evidence	57.07^b^	166.88	23.26, 90.89
Added items	91.00^c^	214.29	47.58, 134.42
Cash	82.86^c^	306.53	20.76, 144.97

*Note*:

Only mean scores with a different superscript differ significantly from each other (*p* < .05).

Analyses of variance utilising a 2 (evidence instruction: absent versus present) × 2 (honesty statement: absent versus present) between‐subjects design were carried out with the six variables reported in Table 1 as dependent variables. None of the interaction effects were significant (all *p*'s > .108), and the univariate effects for the two factors are presented in Table 2.

**Table 2:** Amount of extra money (on top of the actual costs of the items) in £ claimed as a function of honesty instruction and as a function of the providing evidence instruction

Providing evidence instruction									
	Absent			Present					
	*M*	*SD*	CI	*M*	*SD*	CI	*F*	*p*	*d*
Total	348.79	721.84	194.13, 503.46	223.21	322.51	72.16, 381.75	1.223	.273	0.24
Photo + receipt	28.52	100.68	5.62, 51.43	4.35	52.54	−18.61, 27.23	2.202	.141	0.32
Photo	28.69	79.59	8.49, 48.88	15.50	64.54	−4.54, 35.88	0.819	.368	0.18
Receipt	30.28	87.28	12.38, 48.18	2.79	15.29	015.17, 20.65	4.666	.033	0.53
No evidence	59.72	196.13	12.42, 107.02	54.43	133.44	8.96, 103.64	0.010	.919	0.03
Added items	65.44	145.47	3,57, 127.31	116.56	256.18	55.23, 179.08	1.377	.244	0.25
Cash	136.15	422.48	51.60, 220.70	29.58	75.06	−53.84, 115.41	3.060	.084	0.43
Honesty instruction									
Total	442.76	720.28	283.80, 596.46	135.65	274.98	−17.49, 288.73	7.639	.007	0.62
Photo + receipt	26.30	113.40	2.67, 48.97	6.98	20.24	−15.66, 29.69	1.329	.252	0.29
Photo	38.36	82.73	17.57, 58.39	6.48	57.47	−13.61, 26.37	4.826	.031	0.45
Receipt	23.69	84.05	5.14, 41.32	9.67	34.59	−7.93, 27.51	1.112	.294	0.24
No evidence	95.35	137.10	47.67, 143.92	20.36	185.18	−26.28, 67.37	4.945	.029	0.47
Added items	102.77	255.76	40.82, 165.90	79.71	167.13	17.98, 140.48	0.300	.585	0.11
Cash	156.38	426.36	68.80, 239.73	12.45	37.68	17.98, 140.48	5.527	.021	0.62

Regarding the evidence instruction, no difference emerged in the total amount of money claimed on top of the actual costs of the stolen items between those who received the instruction to provide evidence for their claims and those who did not receive this instruction (total score in Table 2, which is the summation of the individual variables). For the individual variables, a significant difference emerged for the receipt‐evidence variable only. Those who were instructed to show evidence claimed less extra cash on top of the actuals costs for the items for which they had a receipt than those who were not instructed to show a receipt.

Regarding the honesty statement, participants who read out the honesty statement claimed less extra money on top of the actual costs of the eight stolen items than those who did not read out such a statement (total score in Table 2). For the individual variables, significant differences emerged for the items without receipts. Those who read out the honesty statement claimed less extra money on top of the actual costs for the photo‐evidence items and no‐evidence items than those who did not read out the honesty statement. In addition, those who read out the honesty statement claimed less cash money than those who did not read out such a statement. These findings showed that the honesty statement factor deterred participants from lying more than the evidence instruction, which supports Hypothesis 1.

### When was the first lie told

For the results presented in this section, the eight participants who did not lie were excluded. The average rank order of the first lie was *M* = 3.17 (*SD* = 2.19, 95% CI [2.71, 3.63]). A 2 (evidence instruction: absent versus present) × 2 (honesty statement: absent versus present) between‐subjects design with the moment the first lie was told as dependent variable revealed a main effect for evidence instruction, *F*(1, 84) = 6.567, *p* = .012, *d* = 0.55. Those who were instructed to show evidence started to lie later (*M* = 3.75, *SD* = 2.35, 95% 95% CI [3.11, 4.39]) than those who were not instructed to show evidence (*M* = 2.59, *SD* = 1.86, 95% CI [1.96, 3.23]). The honesty statement main effect, *F*(1, 84) = 0.124, *p* = .726, *d* = 0.07, and the honesty × evidence instruction interaction effect, *F*(1, 84) = 1.841, *p* = .179, *η*_p_^2^ = 0.021, were not significant.

Table 3 (first column) shows that 28.4% of participants started the interview with lying (lied about the first item they presented). This is significantly fewer than could be expected by chance (50%), *t*(87) = 4.47, *p* < .001, and also significantly fewer than the percentage of lies told across all items (3.29 divided by 8 = 41.2%), *t*(87) = 2.65, *p* = .010, supporting Hypothesis 2. A further 20.5% told their first lie when presenting the second item, and 18.2% told their first lie when presenting their third item. Telling the first lie after the fifth item was rare with only 16% of participants doing that. When we dichotomised this variable and compared the 28.4% of participants who lied when presenting the first item with the remaining participants, Chi‐square tests revealed that the 28.4% of participants who lied when presenting the first item were equally distributed across the two evidence instruction conditions, *X^2^* (*N* = 88) = 1.40, *p* = .35, Φ = 0.17, and the two honesty statement instruction conditions, *X^2^* (*N* = 88) = 1.40, *p* = .35, Φ = 0.17.

**Table 3:** Percentage of participants who lied

Percentage of participants who told				
	First lie on this item	A lie when claiming this item		
	%	*M*	*SD*	CI
Item 1	28.4	28.41	45.36	18.8, 38.0
Item 2	20.5	30.68	46.38	21.1, 40.3
Item 3	18.2	43.18	49.82	32.7, 53.7
Item 4	4.5	30.68	46.38	21.1, 40.3
Item 5	12.5	38.64	49.97	28.4, 48.9
Item 6	8.0	42.05	49.65	31.8, 52.3
Item 7	2.3	43.18	49.82	32.6, 53.7
Item 8	5.7	46.59	50.17	36.1, 57.1

Table 3 also shows how many participants lied when claiming each of the Items 1 to 8 (columns 2–4). In Hypothesis 3, we predicted a linear trend. A polynomial test revealed a significant linear trend, *F*(1, 84) = 9.297, *p* = .003, *η*_p_^2^ = 0.10, with all the other tests being not significant (all *F*'*s* < 3.024, all *p*'*s* > .086). The results showed an upwards trend with an increasing amount of lies told as the interview progressed, supporting Hypothesis 3.

The answers given by the participants in response to the question why they presented the items in the order they did are summarised in Table 4 (they could give several responses so the percentages exceed 100%). Again, we only included participants who told at least one lie (*N* = 88).

**Table 4:** Reported strategies for presenting the items in a certain order

Why did you report items in that order?		
	*N*	%
Random (*n* = 35)/mix in lies and truths (*n* = 18)	53	60.3
To gain trust: Items with receipt first (*n* = 23)/told truths at beginning and lies later (*n* = 11)/price order least to most to gain trust (*n* = 3)	40	41.9
According to gender (Rosie or Jack's items) or location of items in story	14	15.9

Three main strategies emerged: first, using a random approach, which 60.3% of the participants reported to have used; second, to gain trust (41.9%), which was achieved in three ways: report items with receipts first, start with telling the truth and lie later and reporting the cheapest items first. This strategy supports Hypotheses 2 and 3; and third, 15.9% of the participants included items in an order that they thought fit best in their story.

## Discussion

Insurers often ask claimants to produce evidence that the claimed items were actually in the claimants' possession. Although it is understandable that they do so, it is not enough to deter claimants from lying. It had some success, because participants who were asked to show evidence claimed less additional money (on top of the actual costs) for the items for which they did have a receipt than those who were not asked to provide evidence. They also told fewer lies. However, the important figure, the total amount of money claimed, did not differ between those who were asked to provide evidence and those who were not asked to do this. The reason why the evidence instruction did not work is that participants were not inclined to lie about the items for which they had evidence for anyway. They lied more about the stolen items for which there was no evidence than about the stolen items for which there was evidence. And the biggest lies were told by claiming money for items which were, in fact, not stolen and by fabricating that cash was stolen. These findings reveal a clear strategy used by the participants: Provide evidence where possible to make the claim convincing and tell fewer lies perhaps to gain trust; then make a bit of money on stolen items for which there was no evidence and make most of the money on items which were not stolen and on falsely pretending that cash was taken.

Asking claimants before starting to complete the form to read out a statement that they will be honest, actually made them more honest, a finding also obtained by Shu et al. (2012). The significant differences emerged for the items for which there was no evidence available, possibly, as we mentioned earlier, because participants were planning to be honest regarding the items for which there was evidence anyway.

The finding that an honesty declaration at the beginning of a claim leads to more honesty is interesting because it is for insurers (and, for example, tax authorities) very easy to implement. In fact, many already have an honesty declaration but insert this at the end of the claim (in between completing the form and submitting it). Therefore, all insurers need to do is to insert this declaration at the beginning of the claim form. Ariely (2012) reported that the Internal Revenue Service (IRS; US tax collection authority) is reluctant to move the honesty declaration from the end to the beginning of the form. The IRS reported that moving the honesty declaration (and signature) to the beginning would be problematic because the purpose of the signature is to let the claimant declare that the provided information was accurate. Signing a form twice (at the beginning and end) was thought to be ‘confusing’. In our experiment, the participants simply read out a declaration, thus even though nothing was legally signed, just stating they would be honest had a similar effect. Therefore, we consider that this could be a potential solution that the IRS and other companies could consider using in the future.

The present experiment is, to our knowledge, the first experiment in which the timing of the lie was examined. We found that 28.4% of participants lied when presenting the first item. This was a higher percentage than at any other item, but it should be taken into account that for Item 1, everyone had the opportunity to lie or tell the truth, which is not the case for many other items. That is, if someone told four lies, then the first lie could never been told at Items 6, 7 or 8. We also found a trend showing that more lies were told when the interview progressed. These findings suggest that participants prefer to wait with lying. This was according to the hypothesis, which was based on the assumptions that people first would like to make themselves familiar with the situation (e.g. investigator and lie detection tool) and that people would like to gain trust from the investigator. The open‐ended answers indeed supported mainly the latter explanation (gaining trust). Familiarising themselves with the investigator was not mentioned but this may be caused by the fact that the investigator was not visible. The tendency of a large group of participants to start with telling the truth could benefit investigators. For them, it is important to realise that lies are unlikely to be told straight at the beginning of the interview.

## Methodological Consideration

Three methodological issues deserve mentioning. We did not include a condition in the design in which participants read the honesty statement after completing the form (but before submitting it). This is the current practice amongst insurers because it explicitly links the claimant with the form, which is needed for legal reasons. We did not include this condition because Shu et al. (2012) found no effect for this condition in terms of deterrence, which is not surprising. Lying on an insurance claim form is a deliberate act and, once people have carried out that deliberate act, they are unlikely to change their mind about this when they declare that they filled in the form honestly. We did not see merits of replicating the ‘common sense’ null‐effect of Shu et al.

We let participants complete a form on behalf of someone else rather than asking them to pretend that their own home was burgled. We did not ask participants the latter because we expected that some participants would have found this difficult to do (and to lie on the form) for ethical reasons, even for the sake of the experiment. We thought that participants would find it ethically less problematic to lie on behalf of someone else for the sake of the experiment. It could well be that our decision to let participants complete the form on behalf of someone else has affected the frequency and extent of lying. Perhaps people find it easier to justify lying for someone else than for themselves. However, we were not interested in the frequency or extent of lying per se. We were interested in the effect of the two deterrence factors on lying and when the lies would be told. We cannot think of a theoretical reason as to why these issues are affected by completing a form for oneself or on behalf of someone else.

In our experiment, the participants took part in an online, verbal, automated insurance claim setting similar to an automated, verbal, phone call system used by the financial industry (e.g. banks and insurance companies). The question arises whether our findings would be replicated if claimants would fill out a written form, a method also used by the financial industry. This is relevant because someone could argue that written reports feel more distant and therefore make it easier for someone to disengage his or her internal controls (Bandura, Barbaranelli, Caprara, & Pastorelli, 1996). Shu et al. (2012) raised this very issue and therefore used written reports to test the honesty statement hypothesis. They obtained the same findings as we did, suggesting that an honesty statement also works in written statements.
